# Analysis of the expression level and predictive value of CLEC16A|miR-654-5p|RARA regulatory axis in the peripheral blood of patients with ischemic stroke based on biosignature analysis

**DOI:** 10.3389/fneur.2024.1353275

**Published:** 2024-04-12

**Authors:** Jiang-jie Hao, Yuan Liu, Jun-hua Lu, Ying Zhao, Ying Lin, Li-qiu Ma, Ping Xue, Bao-yun Jin, Bei-bei Li, Zheng Zhou, Xin-xin Huang, Ting Liu, Meng-yue Li, Jin-ying Lai, Hong-jun Guan

**Affiliations:** ^1^Department of Public Health, Mudanjiang Medical University, Mudanjiang, China; ^2^Department of Nursing, Mudanjiang Medical University, Mudanjiang, China; ^3^Hongqi Hospital Affiliated to Mudanjiang Medical University, Mudanjiang, China

**Keywords:** ischemic stroke, ceRNA, biomarker, bioinformatics, CLEC16A, miR-654-5p, RARA

## Abstract

**Introduction:**

Ischemic stroke (IS) is a cerebrovascular disease that can be disabling and fatal, and there are limitations in the clinical treatment and prognosis of IS. It has been reported that changes in the expression profile of circRNAs have been found during injury in ischemic stroke, and circRNAs play an important role in the IS cascade response. However, the specific mechanisms involved in the pathogenesis of IS are not yet fully understood, and thus in-depth studies are needed.

**Methods:**

In this study, one circRNA dataset (GSE161913), one miRNA dataset (GSE60319) and one mRNA dataset (GSE180470) were retrieved from the Gene Expression Omnibus (GEO) database and included, and the datasets were differentially expressed analyzed by GEO2R and easyGEO to get the DEcircRNA, DEmiRNA and DEmRNA, and DEmRNA was enriched using ImageGP, binding sites were predicted in the ENCORI database, respectively, and the competitive endogenous RNA (ceRNA) regulatory network was visualized by the cytoscape software, and then selected by MCC scoring in the cytoHubba plugin Hub genes. In addition, this study conducted a case–control study in which blood samples were collected from stroke patients and healthy medical examiners to validate the core network of ceRNAs constructed by biosignature analysis by real-time fluorescence quantitative qRT-PCR experiments.

**Results:**

A total of 233 DEcircRNAs, 132 DEmiRNAs and 72 DEmRNAs were screened by bioinformatics analysis. circRNA-mediated ceRNA regulatory network was constructed, including 148 circRNAs, 43 miRNAs and 44 mRNAs. Finally, CLEC16A|miR-654-5p|RARA competitive endogenous regulatory axis was selected for validation by qRT-PCR, and the validation results were consistent with the bioinformatics analysis.

**Discussion:**

In conclusion, the present study establishes a new axis of regulation associated with IS, providing new insights into the pathogenesis of IS.

## 1 Introduction

Stroke is a type of cerebrovascular disease in which a cerebral blood vessel suddenly ruptures or becomes blocked, resulting in insufficient blood supply to the brain and thus causing severe brain tissue damage and loss of neuronal function. It is one of the leading causes of mortality and morbidity worldwide and is a chronic non-communicable disease that poses a serious threat to the health of the global population. Ischemic stroke (IS) is one of the two main categories of stroke (1), commonly known as cerebral infarction, and refers to the occlusion of the arteries supplying the brain, which leads to necrosis of brain tissue and focal neurological deficits. Ischemic strokes occur every year, and most of them are in the elderly, and can be seriously disabling and fatal (2), causing a heavy medical burden on patients, families and society. According to the data published by the World Health Organization (WHO), the incidence of stroke is getting younger globally, which is a disturbing trend as people’s knowledge about stroke is increasing. Therefore, the prevention and treatment of ischemic stroke should be given sufficient attention to elucidate its pathogenesis as soon as possible and develop solutions to improve the quality of life of the population.

The current diagnosis of stroke relies on clinical symptoms and medical imaging techniques. The most commonly used diagnostic tools for IS are computed tomography (CT) and magnetic resonance (MR) (3). However, CT has poor sensitivity to early ischemic changes in the brain and most patients do not show typical imaging changes in the early stages of the disease, which may miss the optimal time for treatment. Restoration of cerebral blood flow supply is currently the main therapeutic strategy for acute cerebrovascular disease, and early re-establishment of cerebral blood supply by revascularization techniques of intravenous thrombolysis or arterial thrombolysis is the most effective clinical treatment for IS (4). However, there are limitations in the duration of treatment and the application of techniques, and most patients are left with irreversible brain tissue damage with the risk of hemorrhage and ischemia–reperfusion injury (5). Therefore, it is urgent to explore a rapid and accurate biomarker for early diagnosis and prediction of IS, and it is important to further elucidate the pathophysiological mechanisms of ischemic stroke and find timely and effective therapeutic targets.

In recent years, many studies have reported that changes in the expression profiles of noncoding RNAs (ncRNAs) were found during the injury process in ischemic stroke. miRNAs are a class of ubiquitous single-stranded non-coding RNAs that can specifically bind to messenger RNAs (mRNAs) through base complementary pairing, interfering with the translational process and thus inhibiting or altering protein synthesis. Numerous studies have shown that miRNAs, as important mediators of gene regulation, play important roles in IS cascade reactions. Translateral ventricular injection of miR-377 inhibitor in MCAO rats attenuates ischemic brain injury by promoting angiogenesis and inhibiting brain inflammation induced by pro-inflammatory factor release (6). miR-455-5p expression is downregulated in brain tissue and peripheral blood after cerebral ischemia/reperfusion (I/R), and overexpression of miR-455-5p reduces neuroinflammation through inhibition of CCR5 expression, thereby attenuating I/R-induced injury (7). Endothelial miR-15a/16–1 is a negative regulator of cerebral angiogenesis and neurological recovery after stroke (8).

Circular RNA (circRNA) is a unique structure produced by reverse splicing with a high degree of stability, interspecies conservation, and tissue expression specificity, making circRNA an ideal molecular biomarker for diagnosis (9). With the development of bioinformatics analysis methods, many scholars have identified differences in the expression of circRNAs in different organisms and tissues. In a study to identify differentially expressed circRNAs in blood samples from Acute IS patients and healthy controls by circRNA microarray, three circRNAs (circFUNDC1, circPDS5B, and circCDC14A) were up-regulated in AIS patients compared to healthy subjects, and the ROC curve showed an area under the curve (AUC) of 0.875, with a specificity of 91% and a sensitivity of 71.5%, indicating that these 3 circRNAs have diagnostic and prognostic value for AIS (10). The expression of circFOXP1 was significantly reduced in the peripheral blood of AIS patients, and overexpression of circFOXP1 could attenuate post-ischemic brain injury by regulating the STAT3/apoptosis signaling pathway (11). Studies have shown that circRNAs can act as miRNA sponges to regulate gene expression, interact with proteins to perform important biological functions, and encode proteins as translational templates. In recent years, the competitive endogenous RNA (ceRNA) mechanism of circRNAs has been extensively studied. Briefly, circRNAs chelate miRNAs through spongy competition and regulate the inhibitory effect of miRNAs on base complementary pairing at target sites in the untranslated region of messenger RNAs (mRNAs), which in turn regulates the expression of downstream target genes (12). A number of studies have identified a close association between circRNA-mediated ceRNA regulatory networks and ischemic stroke pathophysiological processes (13). A study demonstrated that the expression of circ-HECTD1 and tumor necrosis factor receptor-associated factor 3 (TRAF3) was significantly upregulated in ischemic brain tissues, whereas the expression of miR-133b was downregulated. Knockdown of circ-HECTD1 attenuated neuronal damage induced by cerebral ischemia by targeting binding to miR-133b and inhibiting TRAF3 expression, thereby inhibiting OGD-induced apoptosis and NF-κB activation (14). SNHG15 is upregulated in hypoxic–ischemic mice or cellular models, and inhibition of SNHG15 expression ameliorates ischemia-hypoxia-induced neuronal injury and microglial cell inflammation via the miR-302a-3p / STAT1 / NF-κB pathway (13). Han et al. (15) showed that CircHECTD1 levels were significantly increased in a transient middle cerebral artery occlusion (TMCAO) mouse stroke model, which was validated in plasma samples from AIS patients. By interfering with CircHECTD1 expression using an siRNA approach, MIR142 was released and accompanied by the downstream down-regulation of TIPARP expression, which resulted in the improvement of cerebral infarction by inhibiting astrocyte activation. In summary, circRNA is closely related to the pathophysiological process of ischemic stroke. As the abnormally expressed circRNA molecules are continuously mined for neuroprotective or deleterious effects on IS through the ceRNA regulatory pathway, the specific mechanisms of circRNA and stroke onset, progression and prognosis will be gradually clarified.

**Figure 1:**
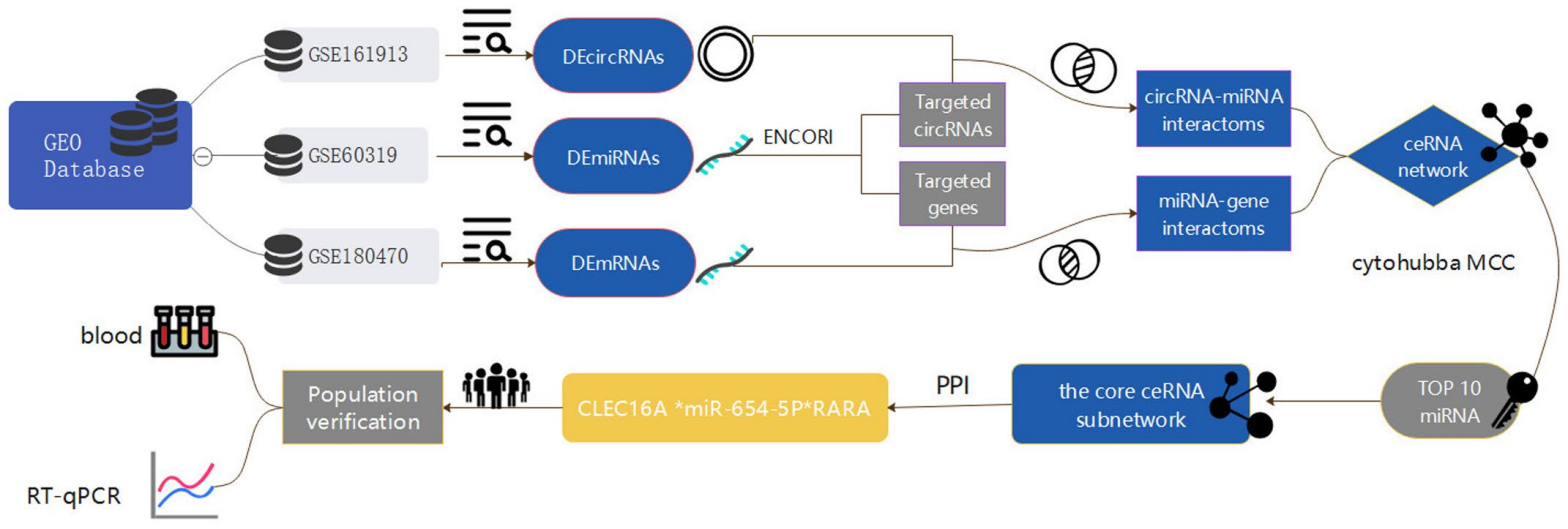
Flowchart of the entire analysis step. In this study, one circRNA dataset (GSE161913), one miRNA dataset (GSE60319) and one mRNA dataset (GSE180470) were retrieved from the Gene Expression Omnibus (GEO) database and included, and the datasets were differentially expressed analyzed by GEO2R and easyGEO to get the DEcircRNA, DEmiRNA and DEmRNA, and DEmRNA was enriched using ImageGP, binding sites were predicted in the ENCORI database, respectively, and the competitive endogenous RNA (ceRNA) regulatory network was visualized by the cytoscape software, and then selected by MCC scoring in the cytoHubba plugin Hub genes. In addition, this study conducted a case-control study in which blood samples were collected from stroke patients and healthy medical examiners to validate the core network of ceRNAs constructed by biosignature analysis by real-time fluorescence quantitative qRT-PCR experiments.

In this paper, we take ischemic stroke as an entry point to reveal the pathogenesis of ischemic cerebrovascular diseases from the perspective of genomics, so as to provide more valuable reference information for the clinic. In this study, we obtained circRNA, miRNA and mRNA expression datasets from the Gene Expression Omnibus (GEO) database, screened the differentially expressed genes and constructed the IS-associated ceRNA network, and then extracted a regulatory axis from them by combining with the Protein–protein interaction (PPI) network analysis, and then verified the gene expression levels through reverse transcription-quantitative polymerase chain reaction (RT-qPCR), and the results of the present study may provide a new idea for elucidating the potential mechanisms underlying the occurrence and development of ischemic stroke. The flow chart illustrating the steps of the whole analysis was shown in Figure 1.

## 2 Materials and methods

### 2.1 Dataset search

All datasets in this study were retrieved from the Gene Expression Omnibus (GEO) database [Home 20 GEO – NCBI (nih.gov)]. The search keywords were “ischemic stroke,” “*Homo sapiens*” and “Non-coding RNA.” The circRNA dataset (GSE161913), miRNA dataset (GSE60319) and mRNA dataset (GSE180470) were finally included in this study. The details of the above three datasets are shown in Table 1.

**Table 1:** Detailed information on the three datasets.

Dataset	Platform	Type	Samples (NC:IS)	Experiment type	Sample	Year	Author
GSE161913	GPL21290	circRNA	4:5	RNA-seq	blood	2021	You Li
GSE60319	GPL19071	microRNA	82:117	array	blood	2015	Pengfei Li
GSE180470	GPL20301	mRNA	3:3	RNA-seq	blood	2021	Yingshuang Wang

### 2.2 Differential expression analysis

Differentially expressed circRNAs, miRNAs and mRNAs were identified under screening criteria. |log2FC| > 2 and *p*-value <0.05 were used as screening criteria. DEcircRNAs and DEmiRNAs were identified by GEO2R [GEO2R – GEO – NCBI (nih.gov)], and DEmRNAs were easyGEO [easyGEO – NCBI GEO's gene expression data analysis and visualization (ubc.ca)] Identification. The results of differential expression analysis for each dataset were visualized by volcano and heat maps in image GP [ImageGP | ImageGP (bic.ac.cn)].

### 2.3 Functional enrichment analysis

To reveal the function of DEmRNAs, we performed functional enrichment analysis including GO classification annotation and KEGG pathway analysis using hiplot.^1^ Statistical significance was defined as *p*-value less than 0.05.

### 2.4 Construction of the ceRNA network

Initially, we used miRNAs as the core to predict the binding sites of circRNAs of DEmiRNAs and mRNAs of DEmiRNAs, respectively, in the ENCORI database [starBase or ENCORI: Decoding the Encyclopedia of RNA Interactomes (rnasysu.com)], which resulted in two files containing all the relationship pairs, DEmiRNA-circRNA pairs and DEmiRNA-mRNA pairs, respectively. We intersected DEmiRNA-predicted circRNAs with DEcircRNAs, and similarly intersected DEmiRNA-predicted mRNAs with DEmRNAs, and finally constructed the ceRNA network. Based on the above analysis, we constructed a ceRNA functional network consisting of circRNA-miRNA-mRNA. For visualization, we used Cytoscape v3.7.2^2^ to draw the ceRNA network.

### 2.5 Protein–protein interaction (PPI) network integration and core subnetwork construction

DEmRNAs were put in the STRING database [STRING: functional protein association networks (string-db.org)] to create a PPI network. Interactions between genes were explored and interaction scores above 0.4 were considered statistically significant. The interaction networks were downloaded and imported into Cytoscape software for visualization.

In order to construct the core sub-network, starting from miRNAs, the genes with the top twenty miRNA differential multiplicity rankings were selected, and genes that could only form a relationship pair with either circ or m were excluded, and finally 11 miRNAs were involved in constructing the ceRNAnetwork, which was done for visualization in the Cytoscape software, and then MCC scoring was performed by cytoHubba plug-in apps Selection of hub genes.

### 2.6 Research design and study population

The aim of this study was to validate the ceRNA regulatory network constructed by bioinformatics analysis in a population by qRT-PCR experiments. We selected 200 ischemic stroke patients admitted to the Department of Neurology of Hongqi Hospital affiliated to Mudanjiang Medical College from November 2021 to June 2023 as cases. The 200 healthy physical examination subjects from the physical examination center of the above hospital during the same period were selected as controls. Complete baseline data of the study subjects were also collect for epidemiologic study. The study was approved by the Ethics Committee of Mudanjiang Medical College and informed consent was obtained from patients or their families.

### 2.7 Blood sample collection

All blood samples were collected on admission and immediately prior to any treatment. 5 mL of fresh blood was collected into EDTA anticoagulated tubes and processed within 30 min if possible. The collected blood was dispensed into individual RNAase-free centrifuge tubes, and each 1 mL of blood was mixed thoroughly with 1 mL of TRIzol Reagent lysate and stored at −80°C in an ultra-low temperature until RNA extraction.

### 2.8 PCR

Total RNA was isolated from blood samples of ischemic stroke patients and healthy controls using the TRIzol reagent. The concentration of RNA was assessed by a Microvolume UV-Vis spectrophotometer (NanoDropOne), and the RNA quality was controlled by the A260/A280 ratio (the eligibility criteria were A260/A280 > 1.8, and total RNA concentration greater than 100 ng/uL). The cDNA was generated by taking 1 μg from the extracted RNA according to the reverse transcription kit from Xinbei (Shanghai) Biotechnology Co. qRT-PCR analysis was performed in an ABI 7500 real-time fluorescence quantitative PCR detection system, and GAPDH and U6 were used for internal normalization. All samples were analyzed in triplicate, and relative gene expression was determined by the 2-ΔΔCT method. qRT-PCR primers were synthesized by Xinbei (Shanghai) Biotechnology Co.

### 2.9 Diagnostic performance assessment

The three RNAs were evaluated for their ability to act as predictors of IS by identifying the pcr-validated stroke-related regulatory axes through univariate and multivariate logistic regression modeling. The diagnostic properties of the three RNAs and their combined predictive ability for IS were assessed by subject operating characteristics (ROC), and the diagnostic accuracy was evaluated by calculating the area under the curve (AUC). The above results were visualized in Graphpad prism (Prism – GraphPad). Statistical significance was defined as *p* < 0.05.

We compared the baseline characteristics of the collected stroke and control groups and performed descriptive analyzes using SPSS 26.0 to determine the distribution of demographic data and influencing factors in the study population. Categorical variables were expressed as percentages and compared using chi-square tests. The most representative variables were selected by univariate logistic regression analysis, and in order to control for confounding bias, the variables from the univariate screening were brought into the model for multivariate logistic regression analysis to explore the influences that had an independent effect on ischemic stroke. ROC curves were used to assess the relationship and predictive value of the influential factors with ischemic stroke.

## 3 Results

### 3.1 Differentially expressed circRNAs, miRNAs, and mRNAs in IS

**Figure 2:**
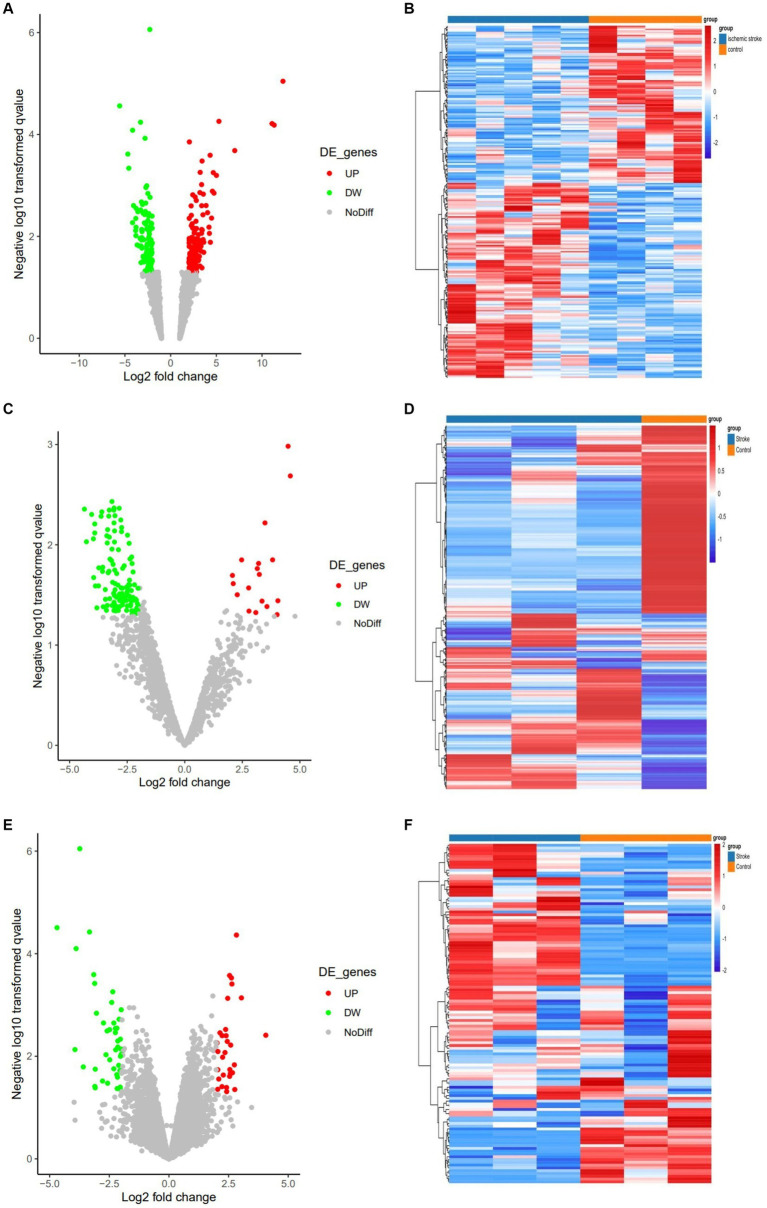
Volcano and heat maps for all circRNAs, miRNAs and mRNAs. (A) Volcano plot of circRNAs. Green and red represent downregulated and upregulated DEcircRNAs, respectively. (B) The heat map of DEcircRNAs. (C) Volcano plot of miRNAs. Green and red represent downregulated and upregulated DEmiRNAs, respectively. (D) The heat map of all DEmiRNAs. (E) Volcano plot of DEmRNAs. (F) The heat map of all DEmRNAs.

Three expression datasets from the GEO database were analyzed in this study. Based on the screening criteria (*p* < 0.05 and | log2 (FC) | > 2), 233 DEcircRNAs (129 up-regulated and 104 down-regulated) were obtained in the GSE161913 dataset, 132 DEmiRNAs (18 up-regulated and 114 down-regulated) in the GSE60319 dataset, and in the GSE180470 dataset 72 DEmRNAs (30 up- and 42 down-regulated) were obtained. The volcano and heat maps of all circRNAs, miRNAs and mRNAs are shown in Figure 2.

### 3.2 Functional enrichment

**Figure 3:**
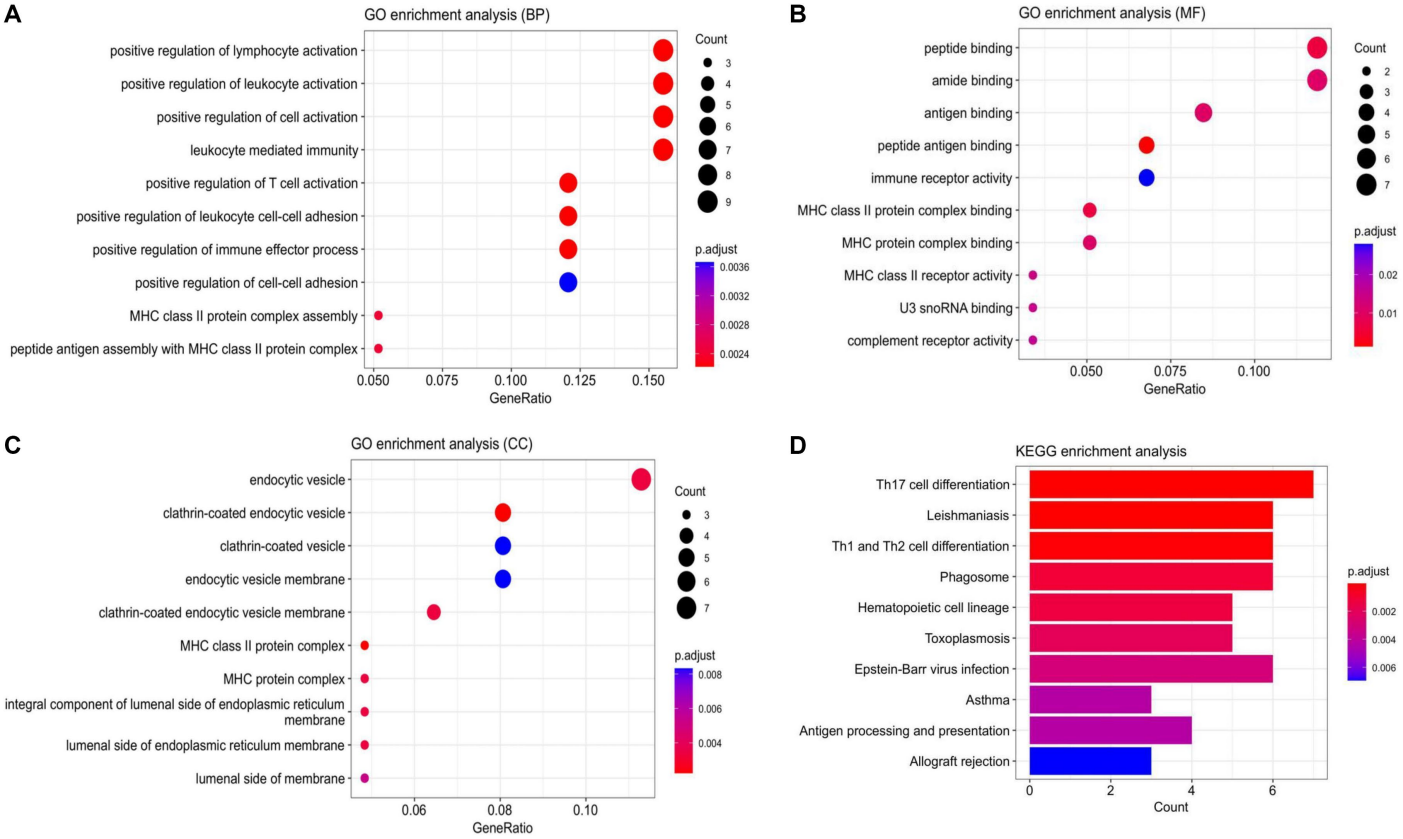
Enrichment analysis results. (A) Scatterplot of BP. (B) Scatterplot of MF. (C) Scatterplot of CC. (D) Bar Plot of KEGG. (BP, biological processes; MF, molecular function; CC, cell component).

Based on the 72 DEmRNAs identified by gene expression profiling in ischemic stroke in the GEO database, their potential biological functions and mechanisms remain unknown. Therefore, it is necessary to further investigate their cellular functions to construct IS-associated ceRNA networks. We performed GO and KEGG analyzes of DEmRNAs, and GO enrichment analyzes were categorized into three main groups: biological processes (BP), cellular components (CC), and molecular functions (MF). These DEmRNAs are mainly enriched in key immune-related biological processes, such as positive regulation of lymphocyte activation, positive regulation of leukocyte activation, leukocyte-mediated immunity, etc. They are mainly involved in a variety of immune-related molecular functions, including peptide-binding, amide-binding, antigen-binding, and immune-receptor activity, and are also involved in a number of specific cellular components such as endocytosis vesicles, lattice protein-encapsulated endocytosis vesicles lattice protein vesicles, endocytosis vesicle membrane, lattice protein-coated endocytosis vesicle membrane, etc. In addition, several significantly enriched KEGG pathways of DEmRNAs were associated with immune responses, including Th17 cell differentiation, Th1 and Th2 cell differentiation, phagolysosomes, Eb virus infection, and hematopoietic cell lineage. The above results suggest that these 72 DEmRNAs may play a role in immune response and inflammatory response. The results of enrichment analysis are shown in Figure 3.

### 3.3 Construction of ceRNA regulatory network

**Figure 4:**
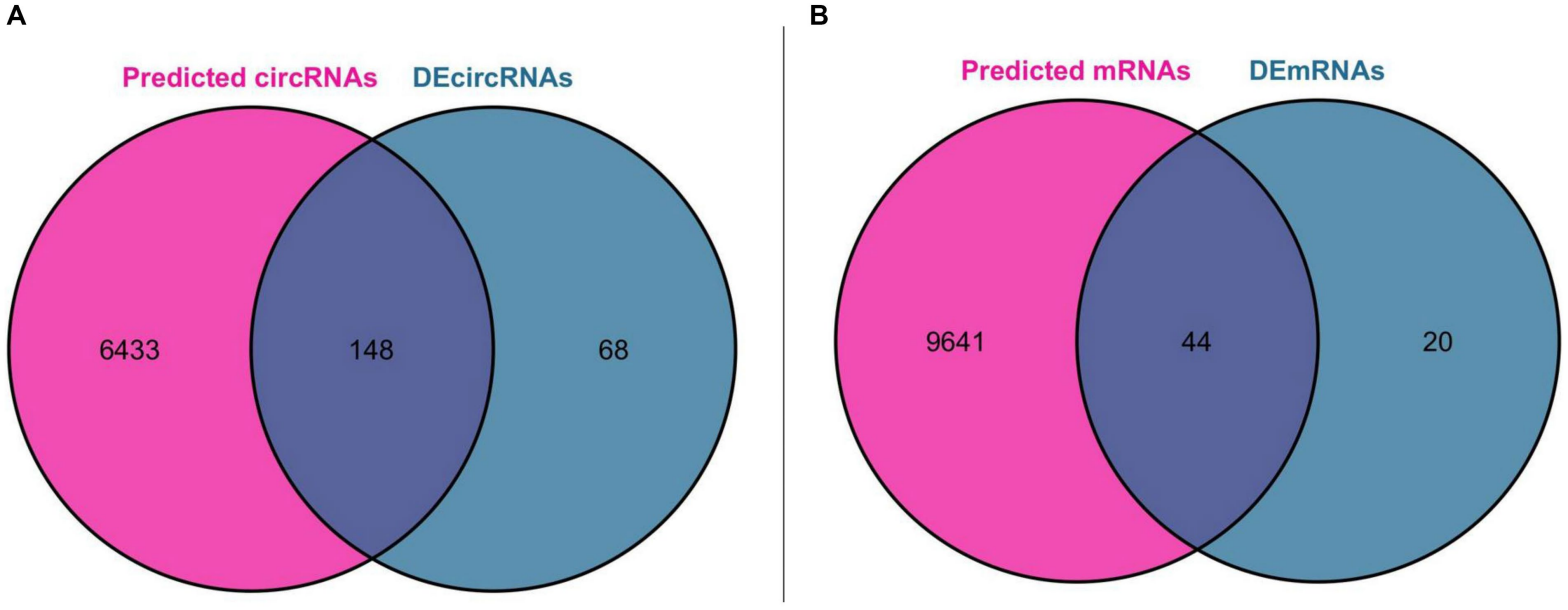
Venn diagram for DEmiRNA binding site prediction. (A) Venn diagram of DEcircRNAs and circRNAs predicted by DEmiRNAs. (B) Venn diagram of DEmRNAs and mRNAs predicted by DEmiRNAs.

Using miRNA as the core, we first predicted the circRNA binding sites of DEmiRNAs in the ENCORI database, and a total of 43 DEmiRNAs found the binding sites of circRNAs, and then predicted the mRNA binding sites of these 43 DEmiRNAs, which resulted in two files containing all the pairs of DEmiRNA-circRNAs (25,534 pairs) and DEmiRNAs-mRNAs (54,248 pairs), respectively. We further obtained miRNA-circRNAs (757 pairs) by intersecting DEmiRNA-predicted circRNAs (6,581 after de-emphasis) with DEcircRNAs (216 after de-emphasis) and filtering out DEmiRNAs that could not be matched with DEcircRNAs. Similarly, the mRNAs predicted by DEmiRNAs (9,685 after de-emphasis) were intersected with DEmRNAs (64 after de-emphasis), and after filtering out the DEmiRNAs that could not be matched with DEmRNAs, further miRNA-mRNAs were obtained (262 pairs). Based on the above analysis, we constructed a ceRNA functional network consisting of circRNA-miRNA-mRNA, which included including 148 circRNAs, 43 miRNAs, and 44 mRNAs. The Venn diagram for DEmiRNA binding site prediction is shown in Figure 4.

### 3.4 PPI analysis

**Figure 5:**
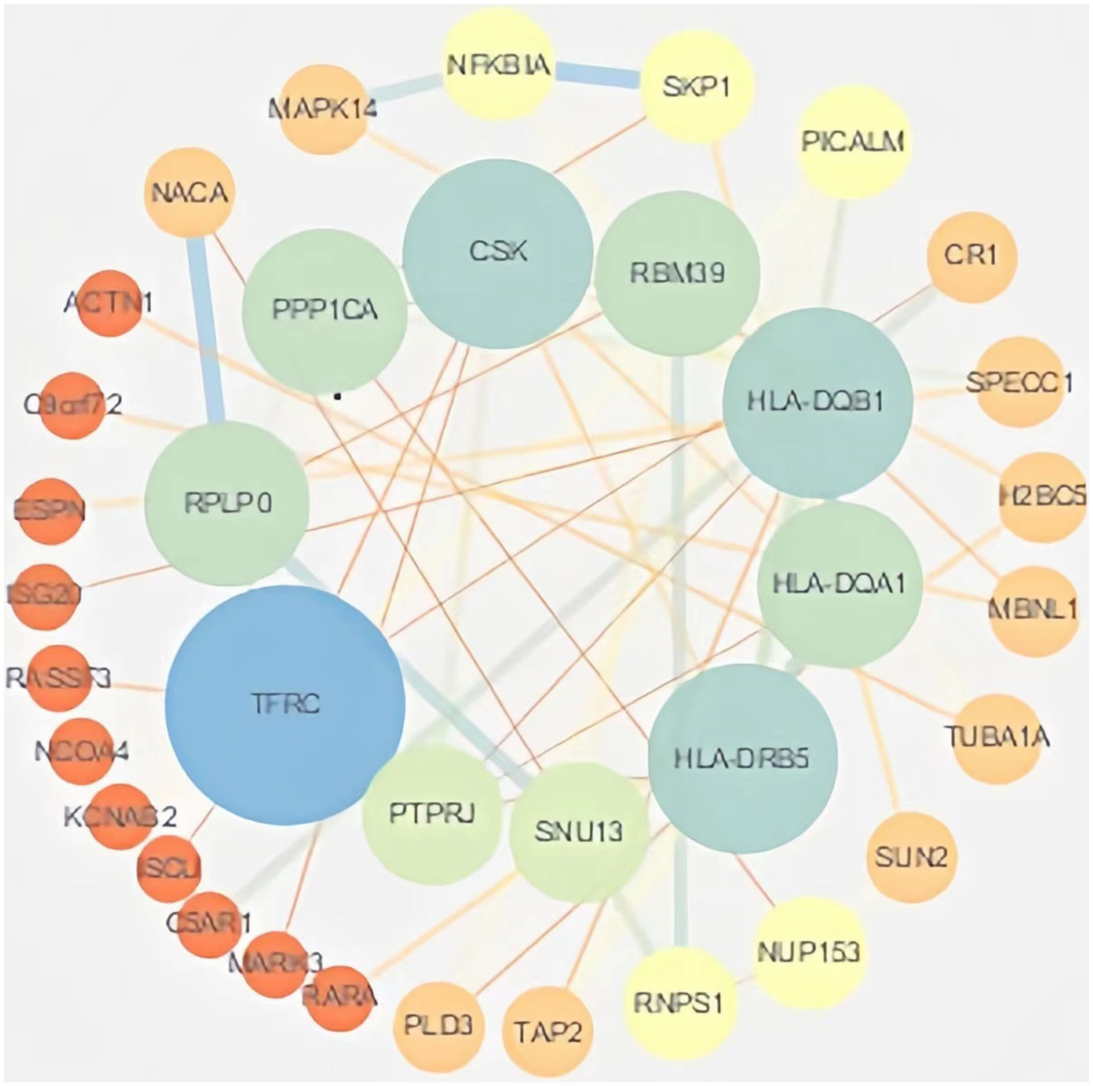
PPI analysis. The PPI network of DEmRNAs constructed by Cytoscape software. Larger nodes indicate higher gene connectivity.

Protein interaction networks are composed of proteins through their interactions with each other to participate in various aspects of life processes such as biological signaling, gene expression regulation, energy and material metabolism and cell cycle regulation. In order to further analyze the interactions of these DEmRNA-encoded proteins in the ceRNA network, the genes obtained from differential analysis were further constructed into a network by using the string database plug-in in Cytoscape software, and after removing the unconnected nodes, the final PPI network included 64 nodes and 52 edges. The results of the PPI analysis are shown in Figure 5.

### 3.5 Core sub-network construction

**Figure 6:**
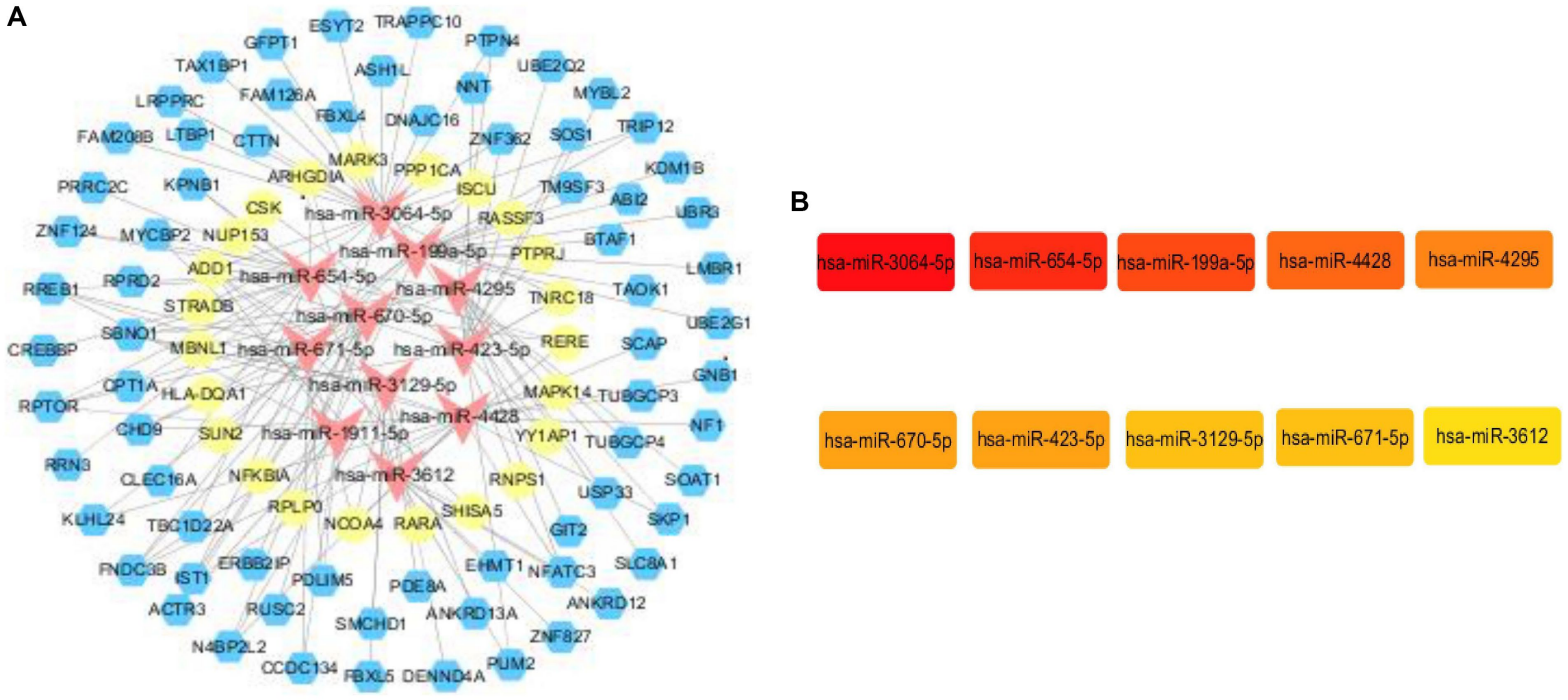
Core subnetwork and key genes. (A) The core sub-network screened from the ceRNA network by the 10 hub genes. Visualization of the ceRNA network. The red nodes represent miRNAs, the blue nodes represent circRNAs, the yellow nodes represent mRNAs. (B) The top 10 hub genes obtained by Cytohubba plugin.

In order to construct the core sub-network, we took miRNAs as the core, ranked the top 20 miRNAs in terms of folding changes, excluded the miRNAs that could only form a relationship pair with either circRNA or mRNA, and finally 11 miRNAs were involved in constructing the ceRNA network, which was visualized in the cytoscape software. Then it was filtered again by MCC score in cytoHubba plugin and TOP10 was visualized, and it was found that the 11 miRNAs that constructed the network ranked according to the folding changes contained TOP10 filtered by MCC score. Therefore, We used the 10 miRNAs screened as core genes to construct the core sub-network. We believe that every candidate gene in the final constructed core sub-network deserves to be paid attention to, but limited by human, material and financial resources and other factors, it is not possible to carry out a comprehensive study of all the genes in the core sub-network, so we choose only one of the regulatory axes among so many candidate genes for validation. For the selection of genes, we are not only concerned about its ranking in a certain algorithm, or fold change, but also whether it can have a direct or indirect connection with our research content. If a gene has some literature in the field we are studying, it is more valuable and experimentally feasible to expand and go deeper in the direction of existing research than to study a gene in a direction it is not involved in. Among them, miR-3064-5p was ranked TOP1 in MCC score. in order to have a further understanding of miR-3064-5p, reference was made to existing studies on it by reviewing the literature. In the reported studies we found that miR-3064-5p plays an inhibitory role in human hepatocellular carcinoma angiogenesis (16). Hypoxia-induced MALAT1 promotes the proliferation and migration of breast cancer cells by sponging miR-3064-5p (17). circCOL6A3/miR-3064-5p/COL6A3 axis can regulate the malignant behaviors of gastric cancer cells (18) and so on. However, there are no relevant studies reporting that the function of miR-3064-5p and the regulatory network it participates in are closely related to ischemic stroke. Considering the experimental feasibility, we focused our attention on miR-654-5p, which ranked TOP2. In a report, not only the comprehensive analysis of miR-654-5p tumor suppressor function, but also the overall biological function of miR-654-5p was explored, and the predicted target genes of miR-654-5p were enriched. The results showed that neuron-related items such as dendritic and forebrain neuronal development and dopaminergic synapses were enriched, suggesting that miR-645-5p may play a key role in neuron-related functions. KEGG pathways are enriched in MAPK-related signaling, PI3K/AKT pathway, T-cell receptor (TCR) signaling, and Ras signaling, and some of the pathways have been confirmed. It has been shown that many genes play a role in ischemic stroke through the MAPK-related signaling pathway, and T-cell receptor (TCR) signaling is associated with inflammatory responses, and neuroinflammation is a key factor in ischemic stroke-induced brain injury. Therefore, we hypothesized that miR-654-5p may play an important role in the physiopathological process of ischemic stroke. In summary, we excluded TOP1 (miR-3064-5p) and selected TOP2 (miR-654-5p) for validation. All upstream and downstream target genes that have a regulatory relationship with this gene were found in the previously constructed core network. miR-654-5p was lowly expressed and therefore screened among the highly expressed circRNAs and mRNAs that form a relationship pair with it. We still resorted to the literature by referring to published articles. In one study, the C → T polymorphism of CLEC16A was found to be significantly associated with the prevalence of myocardial infarction in Japanese individuals, with BMI and glycosylated hemoglobin content in a recessive model, and with serum triglyceride concentration in a dominant model (19). The association of the C → T polymorphism of CLEC16A with myocardial infarction may be partially attributed to the effect of this single nucleotide polymorphism (SNP) on glucose and triglyceride metabolism. The 150 SNPs including CLEC16A detected in this study were selected from the Genome-Wide Association Study of Ischemic Stroke (GWAS) (20). And glucose and triglyceride metabolism are epidemiologically closely related to the occurrence of ischemic stroke, therefore, CLEC16A was linked to ischemic stroke in our study, and this study provides literature support for our genetic screen of regulatory axes. For the selection of mRNAs we still first considered to find the literature support, unfortunately, among the candidate mRNAs, we did not find any of the genes that can be associated with ischemic stroke, and finally we chose the one with a larger multiplicity of differences. and finally a ceRNA regulatory axis(CLEC16A|miR-654-5p|RARA) is selected for validation. The core sub-network and the key genes are shown in Figure 6.

### 3.6 Validation of CLEC16A|miR-654-5p|RARA expression in IS

**Figure 7:**
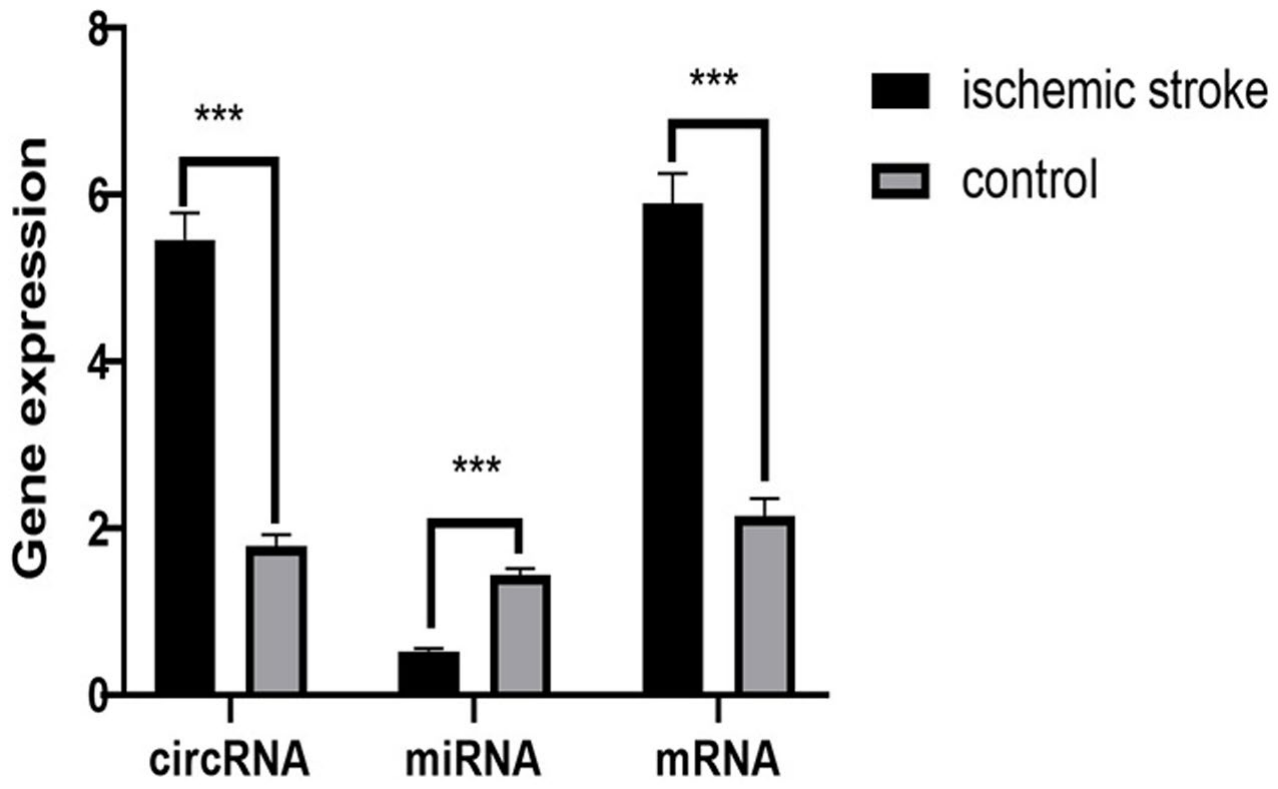
PCR validation results. The circRNA represent CLEC16A, the miRNA represent mir-654-5p, the mRNA represent RARA.

We further validated the final screened ceRNA regulatory axis. qRT-PCR results showed that the expression of CLEC16A and RARA was up-regulated and miR-654-5p was down-regulated, consistent with the results of the BioSense analysis. The PCR validation results are shown in Figure 7.

### 3.7 Baseline information analysis

A total of 400 subjects were included in this study, of which 200 were in the stroke group and 200 in the control group. Comparison of baseline outcomes between stroke and control groups: Differences in hypertension, diabetes mellitus, dyslipidemia, alcohol consumption, and a number of biochemical markers, including total protein, albumin, globulin, total cholesterol, high-density lipoprotein (HDL) cholesterol, LDL cholesterol, and glucose were statistically significant between the two groups (*p* < 0.05), while the differences in triglycerides and smoking were not statistically significant between the two groups (*p* > 0.05). The results of the univariate analysis of ischemic stroke are shown in Table 2.

**Table 2:** Baseline information analysis.

characteristic variable	Case–control study of ischemic stroke		Z/*χ*^2^	*p*
	case group (*n* = 200)	control group (*n* = 200)		
Age (years)	64 (57, 72)	65 (59, 69)	−0.454	0.650
Gender (male)	135 (67.5%)	126 (63%)	0.893	0.345
TP (g/L)	69.6 (65.5, 72.8)	74.15 (71.3, 77.22)	−8.914	<0.001
ALB (g/L)	38.6 (35.55, 41.2)	45.01 (43.12, 46.48)	−13.695	<0.001
GLOB (g/L)	30.6 (28, 33.6)	29.19 (27.32, 32)	−3.086	0.002
TC (mmol/L)	4.60 ± 1.25	4.87 ± 1.09	−2.329	0.020
TG (mmol/L)	1.35 (0.94, 1.90)	1.44 (1.05, 1.97)	−1.195	0.232
HDL-C (mmol/L)	1.00 (0.84, 1.18)	1.25 (1.04, 1.47)	−7.875	<0.001
LDL-C (mmol/L)	2.44 ± 0.79	2.64 ± 0.81	−2.557	0.011
GLU (mmol/L)	6.43 (5.29, 8.37)	4.85 (4.47, 5.37)	−9.936	<0.001
Hypertension (*n*, %)	119 (59.5%)	55 (27.5%)	41.664	<0.001
Asthma (*n*, %)	52 (26%)	17 (8.5%)	21.455	<0.001
Dyslipidemia (*n*, %)	160 (80%)	130 (65%)	11.285	0.001
Smokers (*n*, %)	60 (30%)	47 (23.5%)	2.156	0.142
Alcohol consumption (*n*, %)	100 (50%)	59 (29.5%)	17.547	<0.001

### 3.8 Single factor and multifactor logistic regression analysis of ischemic stroke

A one-way logistic regression equation was constructed, and it was found that total protein, albumin, globulin, total cholesterol, high-density lipoprotein cholesterol, low-density lipoprotein cholesterol, and glucose biochemistry, along with hypertension, diabetes mellitus, dyslipidemia, and alcohol consumption, were the correlates of ischemic stroke (*p* < 0.05).

Multifactorial logistic regression equations were constructed by incorporating factors with *p* < 0.05 in the results of univariate logistic analysis. It was found that the model fit was better when biochemical indicators such as albumin, globulin, total cholesterol, high-density lipoprotein cholesterol, and low-density lipoprotein cholesterol were included in association with hypertension, diabetes mellitus, dyslipidemia, and alcohol consumption (Hosmer-Lemeschow test *p* = 0.263). Among them, albumin (OR = 0.662, 95% CI = 0.602–0.727, *p* < 0.05), total cholesterol (OR = 2.007, 95% CI = 1.079–3.733, *p* < 0.05), HDL cholesterol (OR = 0.153, 95% CI = 0.042–0.553, *p* < 0.05), LDL lipoprotein cholesterol (OR = 0.456, 95% CI = 0.210–0.991, *p* < 0.05), hypertension (OR = 3.086, 95% CI = 1.691–5.632, *p* < 0.05), diabetes mellitus (OR = 4.358, 95% CI = 1.867–10.172, *p* < 0.05), alcohol consumption (OR = 2.274, 95% CI = 1.239–4.174, *p* < 0.05) had a statistically significant effect on ischemic stroke. In contrast, there was no statistically significant effect of globulin (OR = 1.017, 95% CI = 0.945–1.095, *p* > 0.05) and dyslipidemia (OR = 1.044, 95% CI = 0.463–2.354, *p* > 0.05) on ischemic stroke. For the above results, we found that although total cholesterol and LDL cholesterol were statistically significant between ischemic stroke and control group, the case group was overall lower than the control group in the collected biochemical index data, which was in disagreement with the existing studies, therefore, we excluded these influencing factors and included the influencing factors of albumin, HDL cholesterol, hypertension, diabetes mellitus, and alcohol consumption, and once again constructed a multifactorial Logistic regression equation. It was found that the model fit was good (Hosmer-Lemeschow test *p* = 0.152) and the influences of albumin, HDL cholesterol, hypertension, diabetes mellitus and alcohol consumption were statistically significant (*p* < 0.05). Hypertension, diabetes mellitus, and alcohol consumption had ORs greater than 1 and could be considered as independent risk factors for ischemic stroke, whereas albumin and HDL cholesterol had ORs less than 1 and could be considered as protective factors for ischemic stroke. We found a poor model fit (p < 0.05) when three genes in the PCR-validated regulatory axis were used as influences to construct a multifactorial logistic regression model together with other influences. Therefore three genes were included for univariate and multivariate logistic regression analyzes to further assess the association of CLEC16A, miR-654-5p, and RARA with IS. In one-way analysis, the expression levels of CLEC16A, miR-654-5p, and RARA all showed a significant correlation with IS (*p* < 0.001). In multifactorial analysis, the model fit was good (Hosmer-Lemeschow test *p* = 0.063), and CLEC16A (OR = 1.634, 95% CI = 1.429–1.869, *p* < 0.05), mir-654-5p (OR = 0.209, 95% CI = 0.122–0.357, *p* < 0.05), and RARA (OR = 1.301, 95% CI = 1.183–1.431, *p* < 0.05) showed promise as predictive biomarkers for ischemic stroke. The results of the above analysis are shown in Tables 3–6.

**Table 3:** Single factor analysis of ischemic stroke.

	*β*	*p*	OR	95% CI
TP	−0.178	<0.001	0.837	(0.799, 0.877)
ALB	−0.449	<0.001	0.638	(0.586, 0.695)
GLOB	0.080	0.001	1.083	(1.031, 1.037)
TC	−0.203	0.022	0.816	(0.686, 0.971)
HDL-C	−2.411	<0.001	0.090	(0.042, 0.189)
LDL-C	−0.321	0.012	0.725	(0.565, 0.931)
GLU	0.611	<0.001	1.842	(1.554, 2.183)
Hypertension	1.354	<0.001	3.873	(2.546, 5.892)
Asthma	1.330	<0.001	3.782	(2.099, 6.815)
Dyslipidemia	−0.767	0.001	0.464	(0.295, 0.730)
Alcohol consumption	0.871	<0.001	2.390	(1.584, 3.606)

**Table 4:** Multifactorial regression analysis of ischemic stroke.

	*β*	*p*	OR	95% CI
ALB	−0.413	<0.001	0.662	(0.602, 0.727)
GLOB	0.017	0.653	1.017	(0.945, 1.095)
TC	0.697	0.028	2.007	(1.079, 3.733)
HDL-C	−1.877	0.004	0.153	(0.042, 0.553)
LDL-C	−0.785	0.047	0.456	(0.210, 0.991)
Hypertension	1.127	<0.001	3.086	(1.691, 5.632)
Asthma	1.472	0.001	4.358	(1.867, 10.172)
Dyslipidemia	0.043	0.917	1.044	(0.463, 2.354)
Alcohol consumption	0.822	0.008	2.274	(1.239, 4.174)

**Table 5:** Re-analysis.

	*β*	*p*	OR	95% CI
ALB	−0.427	<0.001	0.652	(0.595, 0.716)
HDL-C	−1.107	0.016	0.331	(0.134, 0.817)
Hypertension	1.075	<0.001	2.929	(1.624, 5.285)
Asthma	1.466	0.001	4.331	(1.897, 9.888)
Alcohol consumption	0.841	0.006	2.319	(1.279, 4.202)

**Table 6:** Multifactorial analysis of CLEC16A, mir-654-5p and RARA.

	*β*	*p*	OR	95% CI
CLEC16A	0.491	<0.001	1.634	(1.429, 1.869)
mir-654-5p	−1.567	<0.001	0.209	(0.122, 0.357)
RARA	0.263	<0.001	1.301	(1.183, 1.431)

### 3.9 Analysis of influencing factors on IS diagnostic accuracy

**Figure 8:**
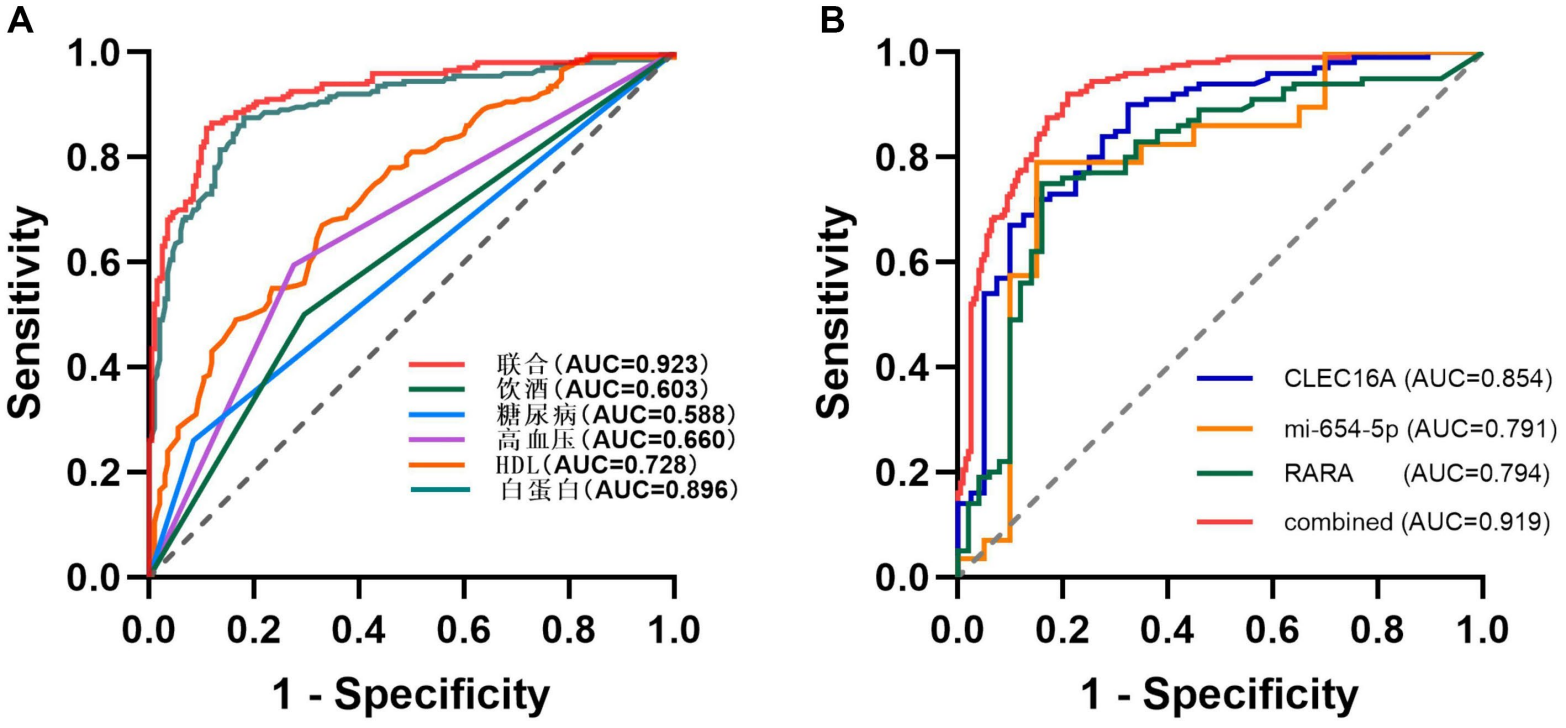
ROC (A) The ROC for IS-related factors. (B) The ROC for CLEC16A, mir-654-5p and RARA.

Using unifactorial and multifactorial joint construction of ROC curves for analysis, ALB (AUC = 0.896), HDL-C (AUC = 0.728), hypertension (AUC = 0.660), diabetes mellitus (AUC = 0.588), and alcohol consumption (AUC = 0.603) had diagnostic potential for IS. The joint analysis showed an area under the curve (AUC = 0.923) with a sensitivity of 86.5% and a specificity of 88.0%, indicating a high diagnostic accuracy for IS. ROC analysis was performed on the PCR-validated regulatory axes, and the area under the curve (AUC) was calculated to assess the diagnostic potential for IS. ROC curve analysis showed that the AUCs of CLEC16A (0.8541), miR-654-5p (0.7913) and RARA (0.7942) were greater than 0.7, which had high diagnostic value. The combination of these three showed significantly higher AUC values for IS identification than for individual RNAs (AUC = 0.919, *p* < 0.001), corresponding to a sensitivity of 92% and a specificity of 79%. The above results are shown in Tables 7 and 8, and 8 Table 7, and Figure 8 shows the ROC curve.

**Table 7:** Analysis of the diagnostic accuracy.

	AUC (95%CI)	*p*	Sensitivity	Specificity	Youden index	Cutoff
ALB	0.896 (0.864, 0.928)	<0.001	0.875	0.820	0.695	42.515
HDL-C	0.728 (0.679, 0.776)	<0.001	0.670	0.670	0.340	1.105
Hypertension	0.660 (0.606, 0.714)	<0.001	0.595	0.725	0.320	
Asthma	0.588 (0.532, 0.643)	0.002	0.260	0.915	0.175	
Alcohol consumption	0.603 (0.547, 0.658)	<0.001	0.500	0.705	0.205	
Combined	0.923 (0.896, 0.950)	<0.001	0.865	0.880	0.745	

**Table 8:** Diagnostic performance analysis of CLEC16A, miR-654-5p and RARA.

	AUC (95%CI)	*p*	Sensitivity	Specificity
CLEC16A	0.854 (0.817, 0.892)	<0.001	0.900	0.675
miR-654-5p	0.791 (0.744, 0.838)	<0.001	0.790	0.850
RARA	0.795 (0.750, 0.841)	<0.001	0.750	0.840
Combined	0.919 (0.892, 0.946)	<0.001	0.920	0.790

## 4 Discussion

At present, for the treatment of IS, the clinic adopts endovascular thrombolysis, drug anticoagulation, surgical thrombolysis, and appropriate neurocellular protection therapy according to different causes and disease processes, and the patient’s condition will improve, but there are still a lot of problems in the process of implementation. Therefore, we also need to explore more therapeutic approaches and implement targeted treatment programs for patients with IS in order to achieve the desired therapeutic outcomes (Table 8).

It has been reported that miRNAs play an important regulatory role in CNS diseases. miRNAs act as gene regulators that can target and regulate the expression of multiple proteins in multiple networks, and in a number of studies miRNAs have been proposed as potential predictive markers for clinical diagnosis of IS and post-stroke recovery (21). In the ceRNA regulatory model, circRNAs can adsorb miRNAs via sponge-like adsorption, thereby inhibiting the regulatory effects of miRNAs on their downstream target genes. This regulation not only enables researchers to have a deeper understanding of the pathophysiology and intermolecular interactions of IS, but also opens up a new continent for the diagnosis and treatment of stroke.

Studies in recent years have shown that there is a correlation between circRNAs and cerebral ischemic events. ceRNAs are currently recognized as one of the major pathways through which circRNAs exert their biological effects, and have been shown to be relevant in animal and cellular experiments as well as in population studies. In one study, this was confirmed not only in animal experiments, but also verified in conjunction with cellular experiments. circPDS5B is significantly upregulated in IS patients and tMCAO mice. Knockdown of circPDS5B, which reduces the area of cerebral infarction and attenuates neuronal damage in tMCAO mice, significantly eliminates the inhibitory effect on OGD/ R-induced human brain microvascular endothelial cells undergoing proliferation, migration, and angiogenesis (22). Plasma levels of circPTP4A2 and circTLK2 expression are significantly elevated in patients with moderate to severe stroke, and ROC curve analysis suggests that they may serve as predictive biomarkers for moderate to severe stroke (23). This study was validated from a population. Many circRNAs have been tapped to play a role in ischemic stroke by mediating the axis of ceRNA regulation. CircUSP36 attenuates stroke-induced brain injury through the miR-139-3p/SMAD3/Bcl2 signaling axis and circUSP36 could be a potential therapeutic target for IS. Although there have been many studies demonstrating the neuromodulatory effects of circRNAs on ischemic stroke, the entire mechanism of IS development has not yet been fully elucidated, and studies on circRNA-mediated ceRNA mechanisms are still lacking (24). Therefore, in this study, we screened 233 DEcircRNAs,132 DEmiRNAs and 72 DEmRNAs based on bioinformatics analysis. By predicting the gene-binding sites and taking the intersections, we constructed a functional network of ceRNAs consisting of circRNA-miRNA-mRNAs, which included 148 circRNAs, 43 miRNAs and 44 mRNAs.

In order to identify potential biological functions, pathway annotation and enrichment analysis of DEmRNAs. These DEmRNAs are mainly enriched in the positive regulation of lymphocyte activation, positive regulation of leukocyte activation and leukocyte-mediated immunity and other related biological processes, and function in peptide-binding, amide-binding and antigen-binding, as well as participate in some specific cellular components, such as endocytosis vesicles and lattice protein-coated endocytosis vesicle membranes. In addition, several KEGG pathways significantly enriched for DEmRNA are associated with immune responses, including Th17 cell differentiation, Th1 and Th2 cell differentiation, phagolysosomes, Eb virus infection, and hematopoietic cell lineage. It has been found that within days or weeks after cerebral ischemia, the immune system disrupts tissue homeostasis, and the inflammatory cascade is immediately activated, with an increase in pro-inflammatory cytokines and an increase in circulating leukocyte aggregates, resulting in blockage within the lumen of the vessel, which further contributes to ischemic injury. The inflammatory response plays a crucial role in all phases of IS pathogenesis. Inflammatory response plays different roles in all stages of IS post-pathogenesis. Th17 and Th1 are pro-inflammatory and Th2 is immunosuppressive. A study suggests that IL-33 promotes th2-type effects after focal stroke and is neuroprotective, but exacerbates systemic immunosuppression after ischemic stroke (25). In another study, Silencing miR-494 expression modulates the HDAC2/STAT4 cascade and attenuates Th1/Th2 imbalance-induced neurological damage (26). An increasing number of studies are being conducted around immune cells and inflammatory cytokines, and intervening in inflammatory immunoregulatory pathways shows potential possibilities in the treatment of cerebral ischemia.

We further combined the MCC scores in the cytoHubba plugin to identify TOP10 as a core gene and constructed a core sub-network, next screened TOP genes one by one, combined with PPI analysis, and finally chose a ceRNA regulatory axis (CLEC16A|miR-654-5p|RARA) for validation. CLEC16A is a member of the C-type lectin family, and it has been shown that CLEC16A plays a key role in the regulation of autoinflammatory responses and biological processes in neurodegenerative pathologies, and has pleiotropic roles in the regulation of autophagy and mitochondrial autophagy (27). In addition CLEC16A may play a role in CNS astrocyte-mediated immune response (28). miR-654-5p has been found to regulate biological processes in a variety of cancers, but the exact function remains unclear. A ceRNA-based study demonstrates that Hsa_circ_0085131 acts as a sponge for miR-654-5p to upregulate autophagy-associated factor ATG7 expression and promote cellular chemoresistance (29). Multiple enrichment analysis of predicted target genes of miR-654-5p revealed that miR-645-5p may play a role in neuron-related functions. Multiple enrichment analysis of the predicted target genes of miR-654-5p revealed enrichment in MAPK-related signaling, PI3K/AKT pathway, T-cell receptor (TCR) signaling, and Ras signaling pathways, suggesting that miR-645-5p may play a role in neuron-related functions (30). Most of the current research on RARA has been on the induction of cellular immune responses through the formation of fusion genes, with assembly with PML to form fusion genes being the most widely studied. However, the specific function of RARA is not yet fully understood, and our study contributes to the elucidation of its mechanism of action.

The expression levels of these three genes in the plasma of IS patients were obtained based on bioinformatics analysis, and the existence of regulatory relationships in the ceRNA network still needs to be confirmed experimentally. In this study, we demonstrate for the first time from population-based blood samples that CLEC16A and RARA are highly expressed and miR-654-5p is lowly expressed in IS patients, consistent with the results of the BioSignal analysis and in accordance with the competing endogenous mechanisms. Thus there may be a ceRNA regulatory relationship between CLEC16A, miR-654-5p, and RARA. Our study provides a new avenue for further elucidation of the potential pathogenesis of IS from the field of transcriptomics, and may provide a new diagnostic and therapeutic option at the molecular level for patients with IS, which is potentially of clinical significance. Furthermore, we determined the diagnostic value of CLEC16A, miR-654-5p, and RARA in IS using binary logistic regression analysis combined with ROC curve analysis, and the combination of all three was superior to the diagnostic efficacy of any one of them for IS and demonstrated relatively high sensitivity (92%) and specificity (79%).

The high rate of disability in IS makes IS patients suffer both physically and psychologically, while the risk of death in IS is also quite high. At present, scholars at home and abroad have done a lot of research on its pathogenesis, but so far it has not been clarified, and there is a lack of effective means to prevent or treat it. Studies have shown that hypertension, dyslipidemia, diabetes mellitus, smoking, and alcohol consumption are the most common risk factors for stroke in China, and that all of these factors can be controlled (31). Therefore, interventions targeting these major risk factors at the individual and population levels are important for stroke prevention. We analyzed the information collected on baseline characteristics and found that multiple influencing factors were significantly different between the stroke and control groups (*p* < 0.05). The results of multifactorial analysis in this study showed that the expression levels of albumin and high-density lipoprotein cholesterol, hypertension, diabetes mellitus, and alcohol consumption were strongly associated with ischemic stroke and could be used as independent influences on ischemic stroke. The risk of IS was nearly 2.929 times higher in patients with hypertension and nearly 4.331 times higher in patients with diabetes mellitus, etc. The results of the study showed that smoking, dyslipidemia, triglycerides and globulins, were not significantly associated with ischemic stroke risk, suggesting that they cannot be used as laboratory markers to predict ischemic stroke. Interestingly, we found that the expression levels of total cholesterol and LDL cholesterol were negatively correlated with IS, and that increased expression levels predicted a reduced risk of ischemic stroke. Therefore, according to our findings, clinical control of hypertension and diabetes, healthy eating habits are essential for the prevention of IS. The results of the analysis of the other indicators diverged from the existing studies, which may be caused by the insufficiently large sample size. Therefore, more information needs to be collected and analyzed in future studies to get more reliable conclusions. Early control of relevant factors and identification of changes in biochemical indicators are important in the prevention of IS occurrence, and targeted treatment and intervention in patients who are already symptomatic can alleviate their condition, improve their standard of living and improve their prognosis.

However, there are limitations to our study. First, the blood samples we collected were all from the same hospital, and a randomized multicenter study is needed to validate these findings. Second, the population sample size for RT-PCR was small and needs to be increased for expression validation. Third, although regulatory genes showed high predictive value in the assessment of IS diagnostic performance, there may be confounding by confounding factors. Fourth, the existence of our proposed ceRNA regulatory network mediating IS needs to be further confirmed in animal models or cellular experiments. Fifth, we predicted a possible role in immune response and inflammatory response only by raw letter analysis, and the specific biological functions played in IS pathogenesis need to be studied in depth.

## Data availability statement

The datasets presented in this study can be found in online repositories. The names of the repository/repositories and accession number(s) can be found at: https://www.ncbi.nlm.nih.gov/geo/, GSE161913; https://www.ncbi.nlm.nih.gov/geo/, GSE60319; https://www.ncbi.nlm.nih.gov/geo/, GSE180470.

## Ethics statement

The studies involving humans were approved by Ethics Committee of Mudanjiang Medical College. The studies were conducted in accordance with the local legislation and institutional requirements. The participants provided their written informed consent to participate in this study. Written informed consent was obtained from the individual(s) for the publication of any potentially identifiable images or data included in this article.

## Author contributions

J-jH: Validation, Visualization, Writing – original draft. YuL: Data curation, Writing – review & editing. J-hL: Resources, Writing – review & editing. YZ: Resources, Writing – review & editing. YiL: Resources, Writing – review & editing. L-qM: Resources, Writing – review & editing. PX: Resources, Writing – review & editing. B-yJ: Validation, Writing – review & editing. B-bL: Validation, Writing – review & editing. ZZ: Software, Writing – review & editing. X-xH: Software, Writing – review & editing. TL: Validation, Writing – review & editing. M-yL: Validation, Writing – review & editing. J-yL: Validation, Writing – review & editing. H-jG: Supervision, Writing – review & editing.

## Footnotes

^1^ https://hiplot.com.cn/

^2^ https://cytoscape.org/
