# Supplementary material for: Analysis of the expression level and predictive value of CLEC16A|miR-654-5p|RARA regulatory axis in the peripheral blood of patients with ischemic stroke based on biosignature analysis
Source: Front Neurol. 2024 Apr 12;15:1353275. doi: 10.3389/fneur.2024.1353275 (PMC11047435; doi:10.3389/fneur.2024.1353275)

Supplementary Material

1 Supplementary Figures and Tables

Figure1 Flowchart of the entire analysis step

In this study, one circRNA dataset (GSE161913), one miRNA dataset (GSE60319) and one mRNA dataset (GSE180470) were retrieved from the Gene Expression Omnibus (GEO) database and included, and the datasets were differentially expressed analyzed by GEO2R and easyGEO to get the DEcircRNA, DEmiRNA and DEmRNA, and DEmRNA was enriched using ImageGP, binding sites were predicted in the ENCORI database, respectively, and the competitive endogenous RNA (ceRNA) regulatory network was visualized by the cytoscape software, and then selected by MCC scoring in the cytoHubba plugin Hub genes. In addition, this study conducted a case-control study in which blood samples were collected from stroke patients and healthy medical examiners to validate the core network of ceRNAs constructed by biosignature analysis by real-time fluorescence quantitative qRT-PCR experiments.

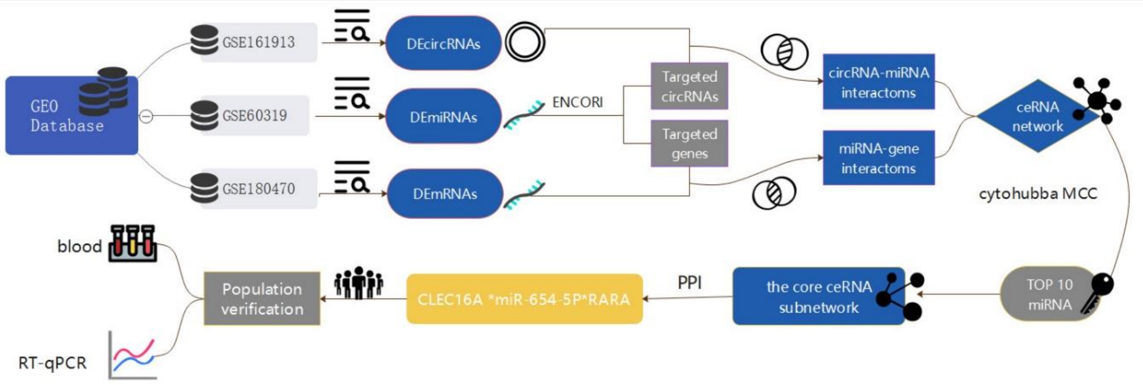

Table 1 Detailed information on the three datasets

dataset	Platform	Type	Samples (NC: IS)	Experiment type	Sample	Year	Author
**GSE161913**	GPL21290	circRNA	4:5	RNA-seq	blood	2021	You Li
**GSE60319**	GPL19071	microRNA	82:117	array	blood	2015	Pengfei Li
**GSE180470**	GPL20301	mRNA	3:3	RNA-seq	blood	2021	Yingshuang Wang

Figure 2 Volcano and heat maps for all circRNAs, miRNAs and mRNAs

(A)Volcano plot of circRNAs. Green and red represent downregulated and upregulated DEcircRNAs, respectively.(B) The heat map of DEcircRNAs.(C) Volcano plot of miRNAs. Green and red

represent downregulated and upregulated DEmiRNAs, respectively.(D) The heat map of all DEmiRNAs. (E) Volcano plot of DEmRNAs.(F) The heat map of all DEmRNAs.

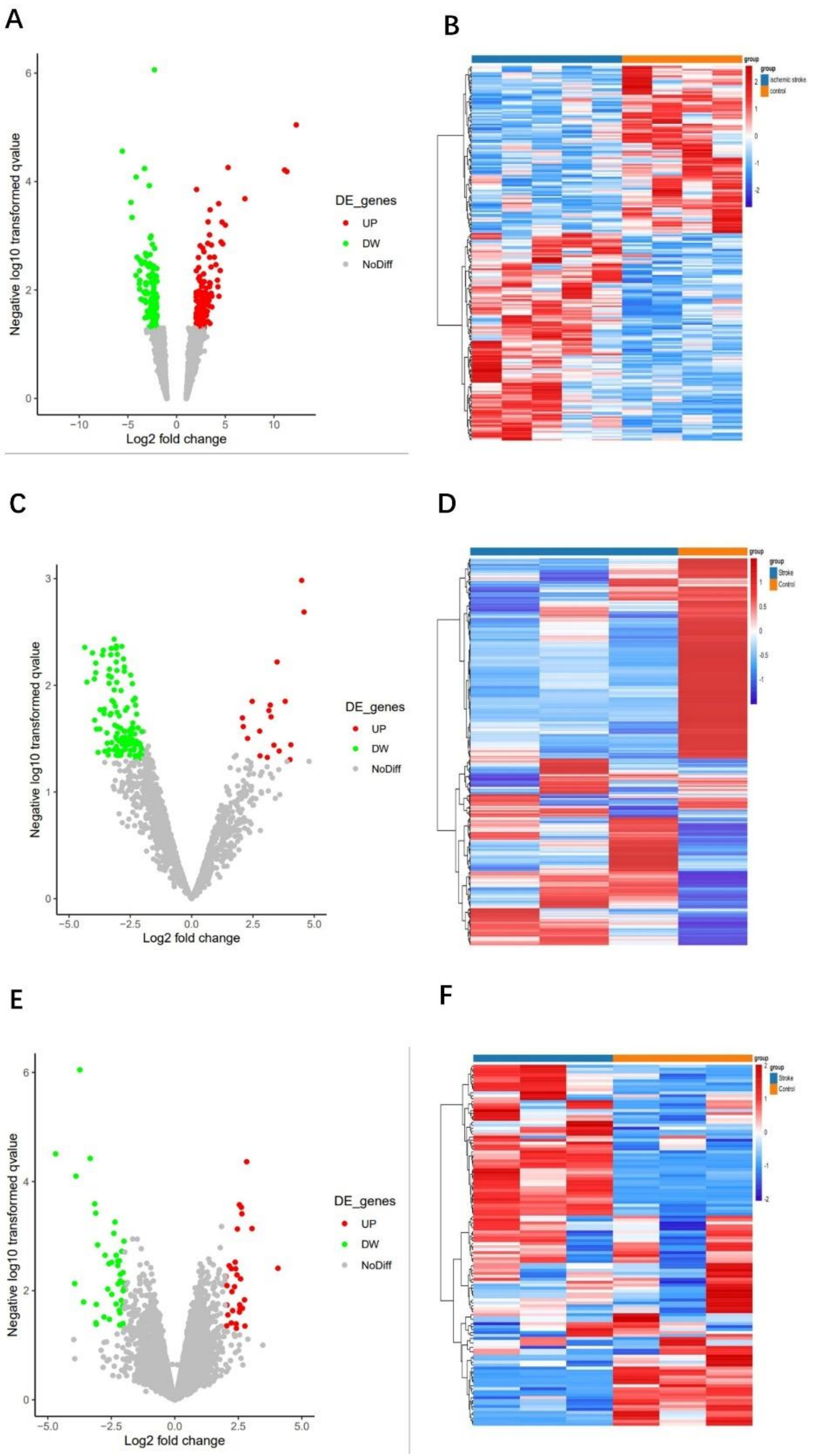

Figure 3 Enrichment analysis results

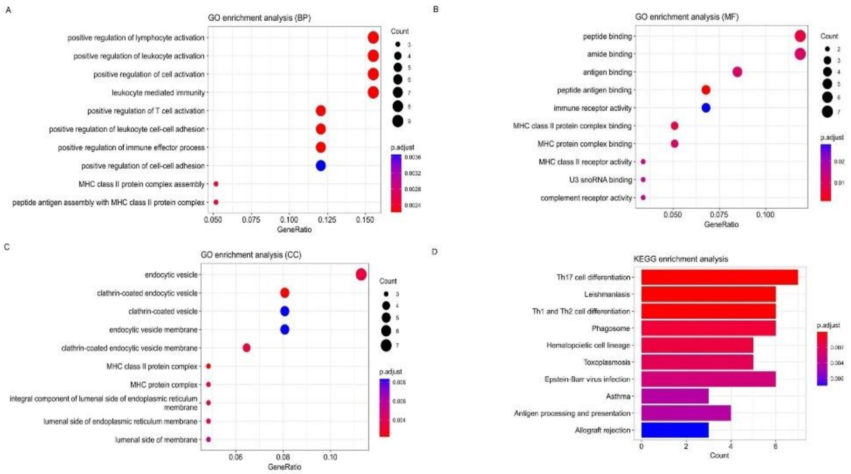

(A) Scatterplot of BP.(B) Scatterplot of MF.(C) Scatterplot of CC.(D) Bar Plot of KEGG. (BP, biological processes; MF, molecular function; CC, cell component).

Figure 4 Venn diagram for DEmiRNA binding site prediction

(A) Venn diagram of DEcircRNAs and circRNAs predicted by DEmiRNAs. (B) Venn diagram of DEmRNAs and mRNAs predicted by DEmiRNAs.

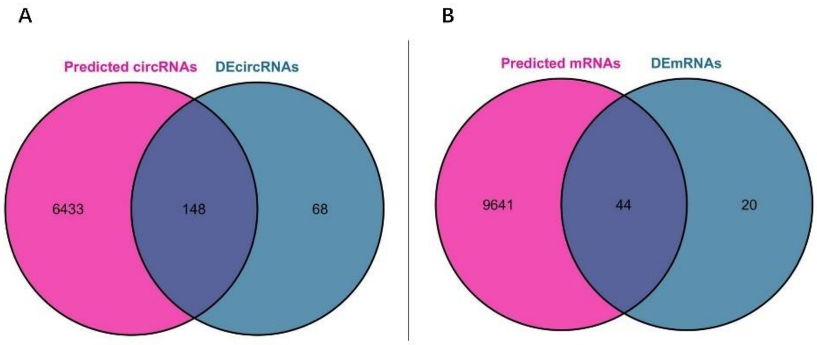

Figure 5 PPI analysis

The PPI network of DEmRNAs constructed by Cytoscape software. Larger nodes indicate higher gene connectivity.

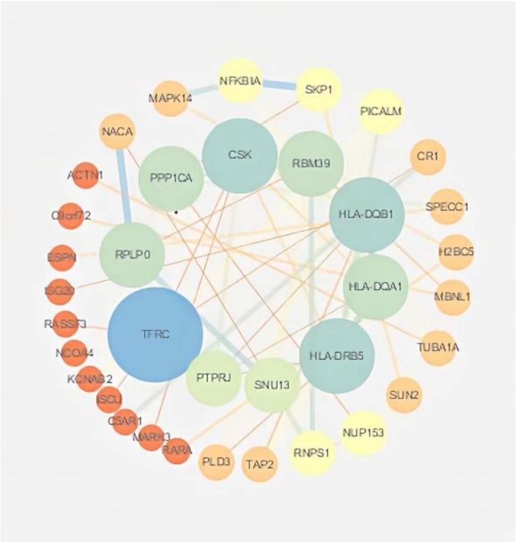

Figure 6 Core subnetwork and key genes

(A) The core sub-network screened from the ceRNA network by the 10 hub genes. Visualization of the ceRNA network. The red nodes represent miRNAs, the blue nodes represent circRNAs, the yellow nodes represent mRNAs. (B) The top 10 hub genes obtained by Cytohubba plugin.

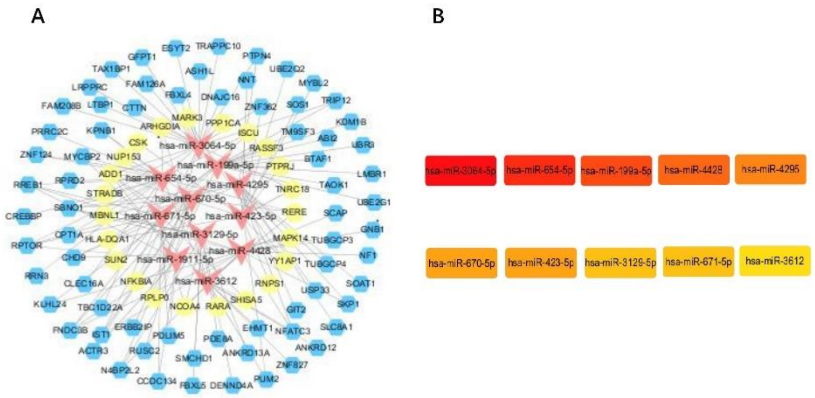

Figure 7 PCR validation results

The circRNA represent CLEC16A, the miRNA represent mir-654-5p, the mRNA represent RARA.

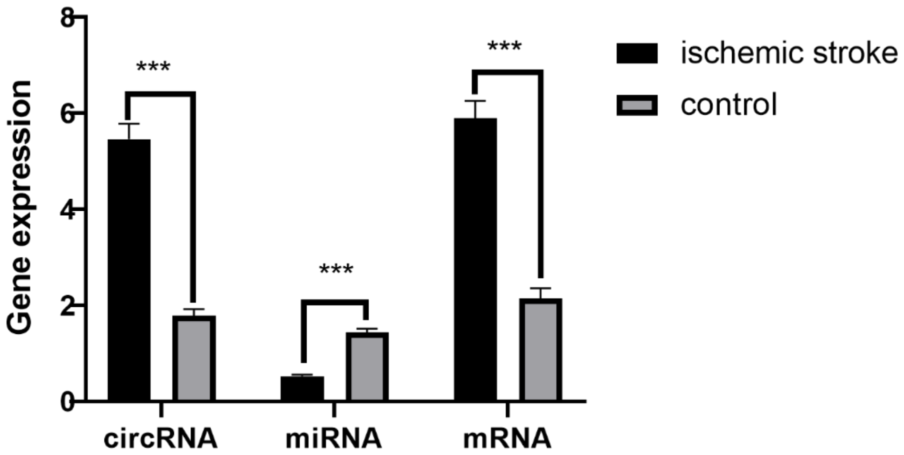

Table 2 Baseline information analysis

characteristic variable	Case-control study of ischemic stroke		Z/χ2	P
	case group（n=200）	control group（n=200）		
**Age (years)**	64（57，72）	65（59，69）	-0.454	0.650
**Gender (male)**	135(67.5%)	126(63%)	0.893	0.345
**TP（g/L)**	69.6（65.5，72.8）	74.15（71.3，77.22）	-8.914	<0.001
**ALB（g/L)**	38.6（35.55，41.2）	45.01（43.12，46.48）	-13.695	<0.001
**GLOB（g/L)**	30.6（28，33.6）	29.19（27.32，32）	-3.086	0.002
**TC（mmol/L）**	4.60±1.25	4.87±1.09	-2.329	0.020
**TG（mmol/L）**	1.35（0.94，1.90）	1.44（1.05，1.97）	-1.195	0.232
**HDL-C（mmol/L）**	1.00（0.84，1.18）	1.25（1.04，1.47）	-7.875	<0.001
**LDL-C（mmol/L）**	2.44±0.79	2.64±0.81	-2.557	0.011
**GLU（mmol/L）**	6.43（5.29，8.37）	4.85（4.47，5.37）	-9.936	<0.001
**hypertension（n，%）**	119(59.5%)	55(27.5%)	41.664	<0.001
**asthma（n，%）**	52(26%)	17(8.5%)	21.455	<0.001
**dyslipidemia（n，%）**	160（80%）	130（65%）	11.285	0.001
**smokers（n，%）**	60(30%)	47(23.5%)	2.156	0.142
**alcohol consumption（n，%）**	100(50%)	59(29.5%)	17.547	<0.001

Table 3 Single factor analysis of ischemic stroke

	β	P	OR	95%CI
**TP**	-0.178	<0.001	0.837	（0.799，0.877）
**ALB**	-0.449	<0.001	0.638	（0.586，0.695）
**GLOB**	0.080	0.001	1.083	（1.031，1.037）
**TC**	-0.203	0.022	0.816	（0.686，0.971）
**HDL-C**	-2.411	<0.001	0.090	（0.042，0.189）
**LDL-C**	-0.321	0.012	0.725	（0.565，0.931）
**GLU**	0.611	<0.001	1.842	（1.554，2.183）
**hypertension**	1.354	<0.001	3.873	（2.546，5.892）
**asthma**	1.330	<0.001	3.782	（2.099，6.815）
**dyslipidemia**	-0.767	0.001	0.464	（0.295，0.730）
**alcohol consumption**	0.871	<0.001	2.390	（1.584，3.606）

Table 4 Multifactorial regression analysis of ischemic stroke

	β	P	OR	95%CI
**ALB**	-0.413	<0.001	0.662	（0.602，0.727）
**GLOB**	0.017	0.653	1.017	（0.945，1.095）
**TC**	0.697	0.028	2.007	（1.079，3.733）
**HDL-C**	-1.877	0.004	0.153	（0.042，0.553）
**LDL-C**	-0.785	0.047	0.456	（0.210，0.991）
**hypertension**	1.127	<0.001	3.086	（1.691，5.632）
**asthma**	1.472	0.001	4.358	（1.867，10.172）
**dyslipidemia**	0.043	0.917	1.044	（0.463，2.354）
**alcohol consumption**	0.822	0.008	2.274	（1.239，4.174）

Table 5 Re-analysis

	β	P	OR	95%CI
**ALB**	-0.427	＜0.001	0.652	（0.595，0.716）
**HDL-C**	-1.107	0.016	0.331	（0.134，0.817）
**hypertension**	1.075	＜0.001	2.929	（1.624，5.285）
**asthma**	1.466	0.001	4.331	（1.897，9.888）
**alcohol consumption**	0.841	0.006	2.319	（1.279，4.202）

Table 6 Multifactorial analysis of CLEC16A, mir-654-5p and RARA

	β	P	OR	95%CI
**CLEC16A**	0.491	<0.001	1.634	（1.429，1.869）
**mir-654-5p**	-1.567	<0.001	0.209	（0.122，0.357）
**RARA**	0.263	<0.001	1.301	（1.183，1.431）

Table 7 Analysis of the diagnostic accuracy

	**AUC(95%CI)**	**P**	**Sensitivity**	**Specificity**	**Youden Index**	**Cutoff**
**ALB**	0.896（0.864，0.928）	＜0.001	0.875	0.820	0.695	42.515
**HDL-C**	0.728（0.679，0.776）	＜0.001	0.670	0.670	0.340	1.105
**hypertension**	0.660（0.606，0.714）	＜0.001	0.595	0.725	0.320	
**asthma**	0.588（0.532，0.643）	0.002	0.260	0.915	0.175	
**alcohol consumption**	0.603（0.547，0.658）	＜0.001	0.500	0.705	0.205	
**combined**	0.923（0.896，0.950）	＜0.001	0.865	0.880	0.745	

Table 8 Diagnostic performance analysis of CLEC16A, miR-654-5p and RARA

	AUC(95%CI)	P	Sensitivity	Specificity
**CLEC16A**	0.854（0.817，0.892）	<0.001	0.900	0.675
**mir-654-5p**	0.791（0.744，0.838）	<0.001	0.790	0.850
**RARA**	0.795（0.750，0.841）	<0.001	0.750	0.840
**Combined**	0.919（0.892，0.946）	<0.001	0.920	0.790

Figure 8 ROC

(A) The ROC for IS-related factors. (B) The ROC for CLEC16A, mir-654-5p and RARA.

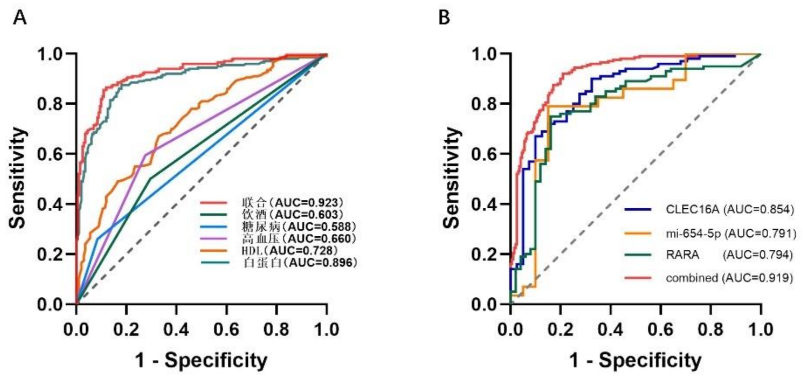

Supplement: Supplementary file 1 [file Data_Sheet_1.pdf]
